# Endometrial Regenerative Cell-Derived Conditioned Medium Alleviates Experimental Colitis

**DOI:** 10.1155/2022/7842296

**Published:** 2022-01-27

**Authors:** Chenglu Sun, Jingpeng Hao, Hong Qin, Yanglin Zhu, Xiang Li, Baoren Zhang, Yafei Qin, Guangming Li, Hongda Wang, Hao Wang

**Affiliations:** ^1^Department of General Surgery, Tianjin Medical University General Hospital, Tianjin, China; ^2^Tianjin General Surgery Institute, Tianjin, China; ^3^Department of Anorectal Surgery, Tianjin Medical University Second Hospital, Tianjin, China

## Abstract

**Background:**

Traditional interventions can play a certain role in attenuating ulcerative colitis (UC), known as one type of inflammatory bowel diseases, but sometimes are not effective. Endometrial regenerative cells (ERCs) have been shown to exert immunosuppressive effects in different models of inflammation, and stem cell-derived conditioned media (CM) have advantages over cell therapy in terms of easy access and direct action. However, whether ERC-CM could alleviate colitis remains unclear and will be explored in this study.

**Methods:**

Menstrual blood was collected from healthy female volunteers to obtain ERCs and ERC-CM. Acute colitis was induced by 3% dextran sodium sulfate (DSS), and ERC-CM was injected on days 4, 6, and 8, respectively, after induction. The disease activity index was calculated through the record of weight change, bleeding, and fecal viscosity during the treatment process. Histological features, macrophage and CD4^+^ T cell in the spleen and colon, and cytokine profiles in the sera and colon were measured. In addition, an *in vitro* lymphocyte proliferation assay was measured by using a CCK-8 kit in this study.

**Results:**

ERC-CM treatment significantly improved the symptoms and histological changes in colitis mice. ERC-CM increased the percentage of Tregs in the spleen and colon but decreased the percentages of M1 macrophages and Th1 and Th17 cells in the spleen and decreased the population of Th17 cells in the colon. In addition, ERC-CM treatment decreased the local expression of TNF-*α*, IL-6, and iNOS in the colon. Furthermore, ERC-CM increased the levels of anti-inflammatory cytokines IL-10 and IL-27 but decreased proinflammatory cytokines IL-6 and IL-17 in the sera. In addition, ERC-CM significantly inhibited ConA-induced mouse lymphocyte proliferation *in vitro*.

**Conclusion:**

The results suggest that ERC-CM can exert similar therapeutic effects as ERCs and could be explored for future application of cell-free therapy in the treatment of colitis.

## 1. Introduction

Since ulcerative colitis (UC), one type of the inflammatory bowel disease (IBD, another type is Crohn's disease) with unknown pathogenesis, was formally established in 1875, medical research on UC has gradually intensified [1]. The pathogenesis of UC is very complex involving genetic factors [2], intestinal microbial factors [3], and intestinal epithelial barrier dysfunction [4]. Innate and adaptive immunity is thought to play a major role in the pathogenesis of UC [5]. Although there are many therapeutic strategies available for UC, such as traditional drugs (mesalamine, glucocorticoids, and thiopurines) [6], biological therapy [7, 8], and diet therapy [9, 10], however, long-term use of immunosuppressive and anti-inflammatory drugs increases the risk of infection and certain malignancies [11]. Unfortunately, many patients with moderate to severe ulcerative colitis inevitably end up needing surgery. In recent years, the treatment of IBD has switched from simply controlling symptoms to normalization of blood markers, complete mucosal healing, and disappearance of symptoms [12]. However, many existing treatment methods cannot meet the needs of all patients. Thus, a novel treatment for UC is urgently needed.

Endometrial regenerative cells (ERCs) from menstrual blood played as a new source of adult stem cells and not only have the potential of self-renewal [13], mesenchymal stem cell- (MSC-) like phenotype [14], and immune modulation [15] but also have the unique advantages of noninvasiveness, relatively unlimited source, strong proliferation ability, and avoiding ethical problems [16, 17]. Our and others' previous studies have demonstrated that ERCs could modulate immune homeostasis in acute hepatitis and other models, such as heterotopic heart transplantation and kidney ischemia reperfusion injury in mice *via* different pathways [18–22]. In ulcerative colitis, ERCs can not only regulate colitis but also be modified for better therapeutic effects [21, 23].

Despite the growing interest in MSCs, some limitations of an MSC-based strategy still remain; for example, most injected cells are arrested by the complex lung network which leads to weak efficiency [24]. More serious is the possibility of thrombosis with cellular administration and other risks such as arrhythmias, ossification, and calcification [25, 26]. In addition, some cryopreservation protocols may make stem cells less functional and viable [27]. All these effects and concerns can be avoided by cell-free preparations. A growing number of studies demonstrated that the therapeutic effects of MSCs are not only due to the cell-to-cell direct contact but also to their secreted cytokines, chemokines, growth factors, extracellular vesicles, and so on. Therefore, due to the characteristics of MSCs and the existence of various limitations, the cell-free treatment strategy is on the rise and has several advantages over cell therapy, as they can be obtained more easily and more economically and can be manufactured, packaged, and transported straightforwardly [28], and the most important thing is that they would not be rejected. All of secreted proteins, EVs and free mitochondria from MSCs, can be found in the conditioned medium (CM) which may exert similar therapeutic function as cell-based treatment. Furthermore, CM-based therapy has been identified to be functional in treating many inflammatory disorders such as hepatic failure and acute lung injury [29, 30]. However, whether ERC-CM could exert the therapeutic effects on experimental colitis remains unclear. Thus, in the present study, the effects of ERC-CM on alleviating colitis were systematically explored in mice.

## 2. Materials and Methods

### 2.1. Isolation and Identification of ERCs

Menstrual blood was collected from healthy female volunteers of childbearing age. All operations towards human being were approved by the Ethics Committee of Tianjin Medical University General Hospital (IRB2021-WZ-116). The protocol for extracting the ERCs was conducted as described previously [19]. Briefly, the menstrual cups were inserted into the vagina for about 4 hours and then removed, and the collected menstrual blood was used for cloudy layer cell collection by the standard Ficoll method and then suspended in Dulbecco's modified Eagle's medium with 10% fetal bovine serum (HyClone) and 1% penicillin/streptomycin and cultured in Petri dishes in an incubator with 37°C and 5% CO_2_ condition. When ERCs grow to the passage 3 (P3), morphology and surface markers (CD90, CD105, CD45, CD79a, and HLA-DR) of ERCs were identified by microscopy and flow cytometry, respectively [22, 23, 31].

### 2.2. Preparation of ERC-CM

ERCs in regular shape and good status of P4-P5 were selected, and the serum-free medium (Yokon, NC0103, China) was replaced until 80% confluency and then collected after 48 h. Next, the cell culture medium, which had been purged of impurities such as cell debris *via* centrifugation (3000 r/min, 20 min), was concentrated 20-fold using an ultrafiltration centrifuge filter unit (Millipore, Billerica, MA, USA) with a molecular weight cut-off value of 3 kDa and then sterilized by filtration through a 0.22 *μ*m centrifugal filter (Millipore, Billerica, MA, USA). The concentrated CM was stored at -80°C for further treatment *in vivo*.

### 2.3. Animals and Experimental Groups

Male mice weighing 22-25 g in this experiment were purchased from National Institutes for Food and Drug Control of China and were placed in standard breeding environment provided with standard diet and water in Animal Care Facility of Tianjin General Surgery Institute. All animal experimental operations were approved by the Institute of Animal Care and Use Committee at Tianjin Medical University General Hospital and performed in accordance with the Guide for the Care and Use of Laboratory Animals (IRB2021-WZ-116).

**Figure 1:**
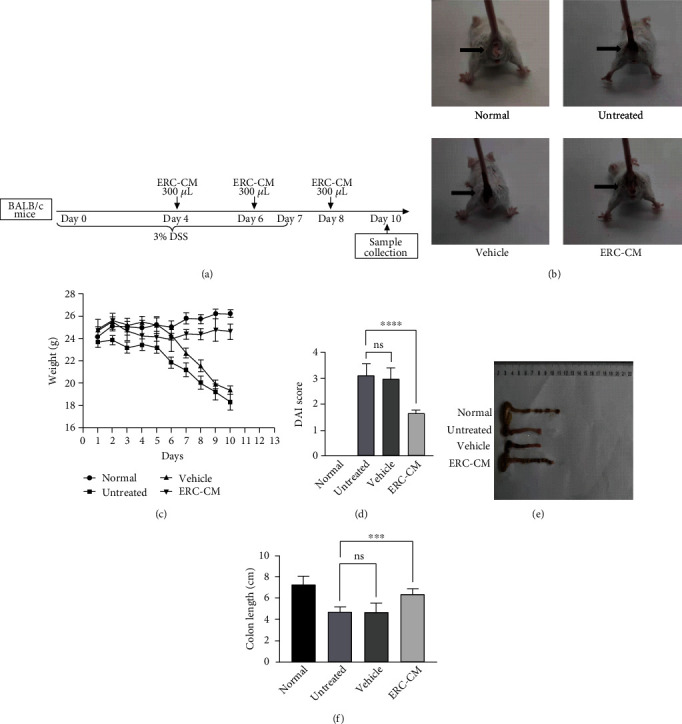
ERC-CM attenuated symptoms in DSS-induced experimental colitis. (a) Study design for the whole process of DSS-induced colitis and interventions. (b) Representative pictures showing bloody stool were taken on the 10th day after DSS induction. The mice in the ERC-CM group were in the best condition than those in other groups. Body weight changes (c) and DAI score (d) of each group of mice were recorded daily. In the ERC-CM group, the weight loss and DAI score were shown lesser than those in other groups. The length of the colon (e, f) in each group was measured and analyzed on day 10 (*n* = 6). Data shown were representative, and the *p* value was determined by one-way ANOVA. ^∗^*p* < 0.05, ^∗∗^*p* < 0.01, ^∗∗∗^*p* < 0.001, and ^∗∗∗∗^*p* < 0.0001.

Acute colitis was induced in BALB/C mice (Figure 1(a)) that received 3% dextran sulfate sodium (DSS, YEASEN, China) in drinking water for 7 days and then consumed normal water for 3 days. Twenty-four mice were randomly divided into four groups (*n* = 6): (I) naive mice drinking normal distilled water for 10 days (normal group), (II) DSS-induced colitis in mice without any treatment (untreated group), (III) DSS-induced colitis mice injected intraperitoneally with 300 *μ*l of normal serum-free medium (days 4, 6, and 8) as the vehicle control (vehicle group), and (IV) DSS-induced colitis mice injected intraperitoneally with 300 *μ*l of ERC-CM (days 4, 6, and 8) (ERC-CM group). Mice were monitored and recorded daily for weight change, stool consistency, and blood in stool for the disease activity index (DAI) calculation based on standard criteria [32]. The mice were sacrificed, and samples (spleen, colon, and sera) were collected at day 10 from the beginning for further analysis.

### 2.4. Pathological Examination

Colon tissue was fixed in 10% formalin for 48 hours and then dehydrated and paraffin-embedded and cut into 5 *μ*m sections for hematoxylin and eosin (H&E) staining. Histopathology scores were evaluated and calculated as in previous studies [33] documented based on the following criteria: (a) inflammation severity: 0 (none), 1 (slight), 2 (moderate), and 3 (severe); (b) depth of injury: 0 (none), 1 (mucosal), 2 (mucosal and submucosal), and 3 (transmural); (c) crypt damage: 0 (none), 1 (basal 1/3 damage), 2 (basal 2/3 damage), 3 (crypt lost, only surface epithelium intact), and 4 (entire crypt and epithelium lost); and (d) percent involvement: 1 (1–25%), 2 (26–50%), 3 (51–75%), and 4 (76–100%).

### 2.5. Immunohistochemistry Staining

Immunohistochemistry staining was used to analyze the expression of TNF-*α*, IL-6, and iNOS in the inflammatory intestine. Firstly, after the slices were hydrated and washed, the slices were submerged in an appropriate EDTA solution and immersed in a 100°C water bath for 15 min to realize antigen retrieval. Secondly, after endogenous peroxidase was eliminated with 3% hydrogen peroxide for 30 min, the nonspecific antibody adsorption was blocked with 10% goat serum for another 30 min, and then, cells were incubated with primary antibodies against TNF-*α* (1 : 400, Abcam, ab92486, UK), IL-6 (1 : 800, Abcam, ab6672, UK), and iNOS (1 : 1000, Abcam, ab283655, UK), respectively, at 4°C overnight. Next, the sections were washed three times with PBS, and a reaction enhancer was added dropwise and incubated for 20 min at room temperature and then incubated with an anti-rabbit IgG antibody (Zhongshan Jinqiao, PV-9000, China) for another 20 min at room temperature. Finally, the sections were incubated with freshly prepared DAB solution (Zhongshan Jinqiao, ZLI-9018, China) after washing, and hematoxylin was added dropwise for nuclear staining and then scanned for image analysis. ImageJ software was used to analyze the image.

### 2.6. Preparation of Lamina Propria Mononuclear Cells and Splenocyte Suspension

Isolation of murine lamina propria mononuclear cells from colonic tissue was conducted under the guideline as described [34, 35]. Briefly, mucus and epithelial cells were removed from fresh colon specimens in successive steps with dithiothreitol (DTT, Solarbio, D8220, China) and ethylene diamine tetraacetic acid (EDTA, Solarbio, E8040, China), followed by digestion with collagenase IV (400 U/ml, Solarbio, C8160, China) and deoxyribonuclease I (0.15 mg/ml, Solarbio, D8071, China) for 90 min at 37°C. The cell precipitate was resuspended with 4 ml 40% Percoll (Cytiva, US), and then, 2.5 mL 80% Percoll was slowly added to the bottom of the centrifuge tube. LPMCs were obtained by centrifugation at 1000 g density gradient for 20 min and then washed with PBS.

Mouse spleens were ground separately, and splenocyte suspension was obtained after lysis of red blood cells. After washing twice, the cell concentration was adjusted to 1 × 10^7^/ml with PBS solution.

### 2.7. Flow Cytometry Analysis

Different immune cells are identified using flow cytometry. All flow cytometry monoclonal antibodies and reagents used in this experiment were purchased from either BioLegend or eBioscience company, mainly including Zombie NIR™ Dye (Dead/Live reagent), anti-mouse CD4 (FITC-labelled), IFN-*γ* (PE-labelled), IL-17 (Percp-labelled), CD25 (PE-labelled), Foxp3 (APC-labelled), CD11b (FITC-labelled), F4/80 (APC-labelled), and CD86 (Percp-labelled) to detect Th1 cells (CD4^+^IFN-*γ*^+^), Th17 cells (CD4^+^IL-17a^+^), Tregs (CD4^+^CD25^+^Foxp3^+^), and M1 macrophages (CD11b^+^F4/80^+^CD86^+^) in the spleen and/or intestine. Extra- and intracellular staining protocols were carried out as previously described [21]. Furthermore, to accurately identify Th1 and Th17, splenocytes and colonic LPMCs were firstly coincubated with stimulators for 6 hours, respectively, followed by fluorescent antibody staining.

### 2.8. Enzyme-Linked Immunosorbent Assay (ELISA)

After the mice were sacrificed on day 10 after DSS induction, the blood was collected to obtain the sera. The levels of IL-10, IL-27, IL-17, and IL-6 in the sera were used to assess the inflammatory status and were measured using corresponding ELISA kits (DAKEWE, Beijing, China), and details were performed according to the manufacturer's instructions.

### 2.9. *In Vitro* Proliferation Assay

The proliferation of lymphocytes in response to ConA was measured by using the CCK-8 kit. Briefly, BALB/c splenocyte suspension (3 × 10^5^ cells/well) was cultured with ConA (20 *μ*g/ml) in the presence of ERC-CM at indicated concentrations. The cultures were incubated for 72 h, 10 *μ*l of CCK-8 was then added to each well before the end of culture, and OD_450 nm_ was recorded.

### 2.10. Statistics

Data shown in this study were expressed as mean ± SD, and the differences among multiple groups were analyzed using one-way analysis of variance (ANOVA); GraphPad Prism 8 software was applied. Throughout the text, figures, and legends, the following terminologies are used to denote statistical significance: ^∗^*p* < 0.05, ^∗∗^*p* < 0.01, ^∗∗∗^*p* < 0.001, and ^∗∗∗∗^*p* < 0.001.

## 3. Results

### 3.1. Characterization of ERCs

**Figure 2:**
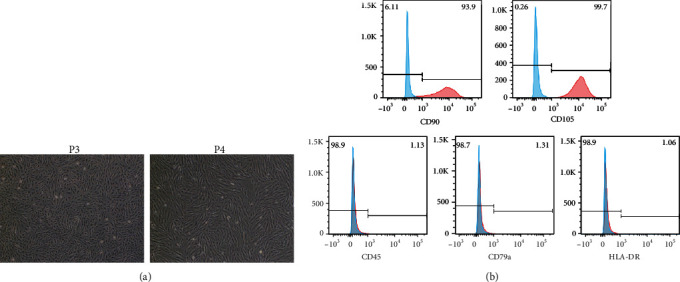
Characterization of ERCs. (a) Cell morphology of ERCs at P3 and P4. (b) Flow cytometry analysis of ERCs. Surface markers CD90, CD105, CD45, CD79a, and HLA-DR were detected.

At P3, ERCs exhibited spindle-shaped, fibroblast-like morphology and colony-forming abilities (Figure 2(a)). ERCs measured by flow cytometry showed high expression of CD90 and CD105 and low expression of CD45, CD79a, and HLA-DR, which are consistent with MSCs' phenotype (Figure 2(b)).

### 3.2. ERC-CM Attenuated Symptoms in DSS-Induced Experimental Colitis

BALB/C mice suffered from severe colitis induced by DSS (Figure 1(a)) characterized by weight loss, bloody diarrhea, and increased general DAI by drinking distilled water containing 3% DSS (Figures 1(b)–1(d)). The colons collected from different groups exhibited different lengths, and the colon in the ERC-CM group seems much longer than that in untreated and/or vehicle groups (ERC-CM group *vs.* untreated group, *p* < 0.001; ERC-CM group *vs.* vehicle group, *p* < 0.001). This result implied that ERC-CM could significantly attenuate the colon's shortening (Figures 1(e)–1(f)).

### 3.3. ERC-CM Significantly Alleviated Colon Damage in Colitis

**Figure 3:**
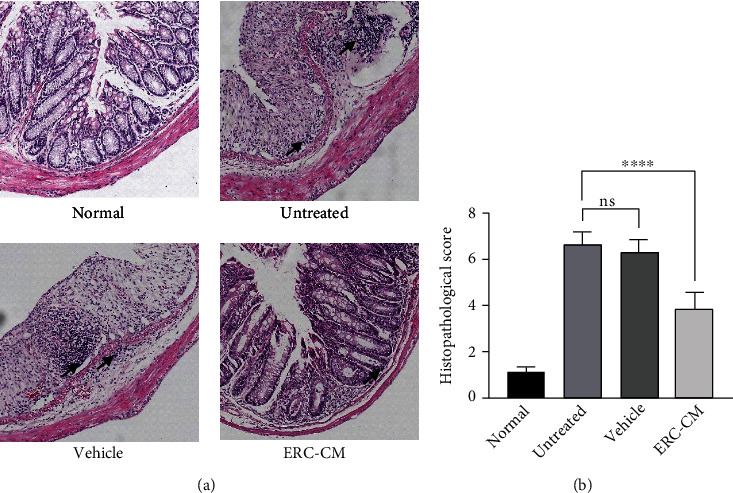
ERC-CM significantly alleviated colon damage in colitis. (a) Representative photomicrographs (100x, hematoxylin and eosin staining) of histological sections of the colon from each group. (b) Histopathology scores were evaluated and calculated as in previous studies [33]. The *p* value was determined by one-way ANOVA. ^∗^*p* < 0.05, ^∗∗^*p* < 0.01, ^∗∗∗^*p* < 0.001, and ^∗∗∗∗^*p* < 0.0001.

Histopathological changes were assessed by hematoxylin/eosin staining of colonic sections. Mucosal structural disorders, inflammatory cell infiltration, epithelial cells, and structural disruption of the crypt were very intense in the untreated group and vehicle group, whereas these changes were significantly attenuated in the ERC-CM treated group. Histopathological scores were used to assess the damage, and scores in the ERC-CM group were significantly lower than those in the untreated and/or vehicle group (ERC-CM group *vs.* untreated group, *p* < 0.0001; ERC-CM group *vs.* vehicle group, *p* < 0.0001) (Figures 3(a) and 3(b)).

### 3.4. ERC-CM Decreased the Percentages of Th1 Cells, Th17 Cells, and M1 Macrophages but Increased the Percentage of Tregs in the Spleen of DSS-Induced Colitis in Mice

**Figure 4:**
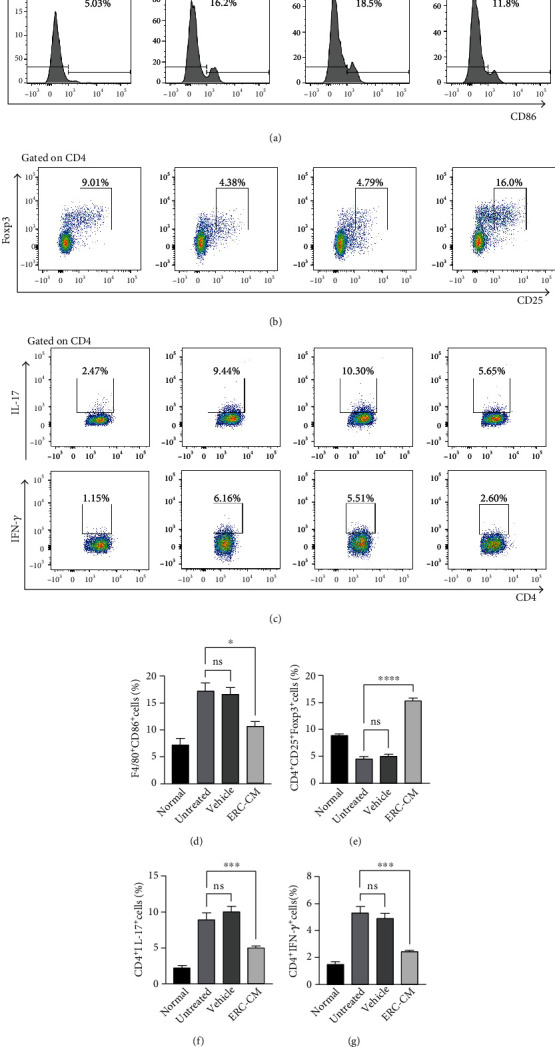
ERC-CM decreased the percentages of Th1 cells, Th17 cells, and M1 macrophages but increased the percentage of Tregs in the spleen of colitis mice. Splenocytes were collected on day 10 after DSS induction. To accurately identify the subpopulation of Th1 and Th17 cells, splenocytes were firstly incubated with a cell stimulation cocktail for 6 hours before being stained with fluorescent antibodies. Percentages of F4/80^+^CD86^+^ M1 macrophages (a), representative dot plots of CD4^+^CD25^+^Foxp3^+^ Tregs (b), CD4^+^IL-17^+^Th17 cells (c), and CD4^+^IFN-*γ*^+^ Th1 cells (d) were shown. Data shown were representative, and the *p* value was determined by one-way ANOVA. ^∗^*p* < 0.05, ^∗∗^*p* < 0.01, ^∗∗∗^*p* < 0.001, and ^∗∗∗∗^*p* < 0.0001.

To determine the immunomodulatory effects of ERC-CM, splenocytes were prepared and stained for analysis. As shown in Figures 4(a) and 4(c), percentages of M1 macrophages, Th1 cells, and Th17 cells were increased in the untreated group and significantly decreased after ERC-CM treatment (Figure 4(d), M1: ERC-CM group *vs.* untreated group, *p* < 0.05; ERC-CM group *vs.* vehicle group, *p* < 0.05; Figure 4(g), Th1: ERC-CM group *vs.* group, *p* < 0.001; ERC-CM *vs.* vehicle, *p* < 0.001; Figure 4(f), Th17: ERC-CM *vs.* untreated, *p* < 0.001; ERC-CM *vs.* vehicle, *p* < 0.001). Furthermore, Treg populations showed a decreasing trend in the untreated and/or vehicle groups but an obvious increase after ERC-CM treatment (ERC-CM group *vs.* untreated group, *p* < 0.0001; ERC-CM group *vs.* vehicle group, *p* < 0.0001) (Figures 4(b) and 4(e)). This indicated that ERC-CM successfully performs an immunomodulatory function.

### 3.5. ERC-CM Decreased the Percentage of Th17 but Increased the Percentage of Tregs in the Colon

**Figure 5:**
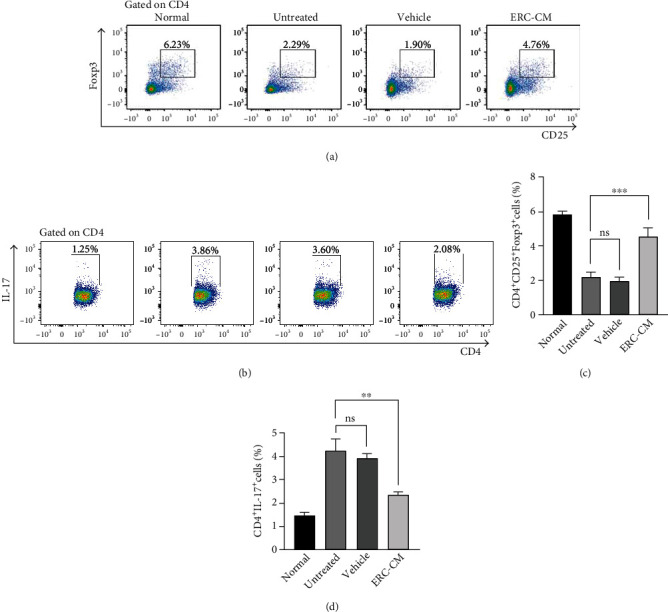
ERC-CM decreased the percentage of Th17 cells but increased the percentage of Tregs in the colon. Representative dot plots of CD4^+^CD25^+^Foxp3^+^ Tregs (a), CD4^+^IL-17^+^ Th17 cells (b) in the lamina propria. Percentage of CD4^+^CD25^+^Foxp3^+^ Tregs (c) and CD4^+^IL-17^+^ Th17 cells (d). Data shown were representative, and the *p* value was determined by one-way ANOVA. ^∗^*p* < 0.05, ^∗∗^*p* < 0.01, ^∗∗∗^*p* < 0.001, and ^∗∗∗∗^*p* < 0.0001.

UC has been shown to be mediated by excessive activation of Th17 cells and deficiency of Tregs [32]. To this end, the expressions of Th17 and Tregs in CD4^+^T cells were analyzed by flow cytometry. As expected, the accumulation of Th17 cells in the lamina propria was significant in the untreated group of mice compared to the normal group of mice and relatively less in the treated group (ERC-CM group *vs.* untreated group, *p* < 0.01; ERC-CM group *vs.* vehicle group, *p* < 0.01) (Figures 5(b) and 5(d)). In contrast, the number of Tregs in the lamina propria was abnormally downregulated in the untreated group compared to the normal group of mice and significantly increased in the treated group (ERC-CM group *vs.* untreated group, *p* < 0.001; ERC-CM group *vs.* vehicle, *p* < 0.001) (Figures 5(a) and 5(c)). Based on this observation, the regulation of Th17 cells and Tregs in the lamina propria by ERC-CM may be associated with the improvement of colitis.

### 3.6. ERC-CM Decreased Proinflammatory Cytokine Levels and Macrophage Infiltration in the Colon

**Figure 6:**
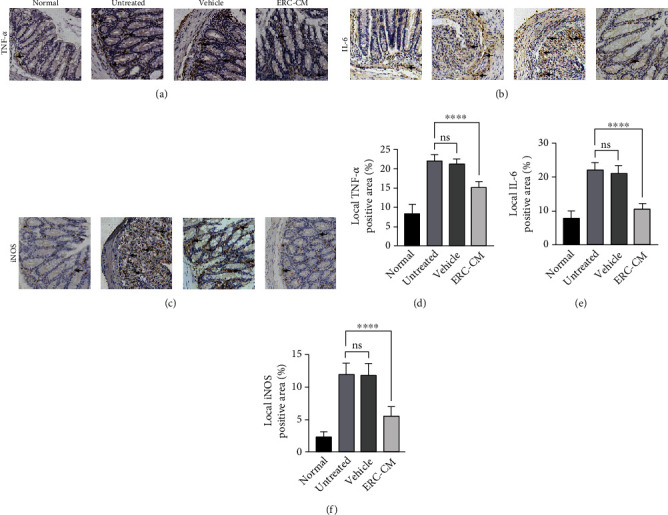
ERC-CM decreased the level of proinflammatory cytokines and macrophage infiltration in the colon. Inflammatory factor levels and macrophage infiltration in the colon were assessed by immunohistochemical staining. Specifically, TNF-*α*, IL-6, and M1 macrophage infiltration were detected by iNOS staining. Images of IHC staining (200x) for the mouse colon (a–c) and quantitative data of cell counts for each group (d–f) are shown. The *p* value was determined by one-way ANOVA. ^∗^*p* < 0.05, ^∗∗^*p* < 0.01, ^∗∗∗^*p* < 0.001, and ^∗∗∗∗^*p* < 0.0001.

To detect the regulatory effect of ERC-CM on proinflammatory cytokines and inflammatory cells in the colon, we detected the expression of TNF-*α*, IL-6, and M1 macrophages by immunohistochemistry. As shown in Figures 6(a) and 6(b), TNF-*α* and IL-6 were significantly increased in the untreated group but were decreased after ERC-CM treatment (Figure 6(d), TNF-*α*: ERC-CM group *vs.* untreated group, *p* < 0.0001; ERC-CM group *vs.* vehicle group, *p* < 0.0001; Figure 6(e), IL-6: ERC-CM group *vs.* untreated group, *p* < 0.0001; ERC-CM group *vs.* vehicle group, *p* < 0.0001). Similarly, as shown in Figures 6(c) and 6(f), M1 macrophages (iNOS^+^ cells) showed a significant infiltration in the untreated group and were decreased after ERC-CM treatment (ERC-CM group *vs.* untreated group, *p* < 0.0001; ERC-CM group *vs.* vehicle group, *p* < 0.0001).

### 3.7. ERC-CM Modulated Cytokine Level in the Sera

**Figure 7:**
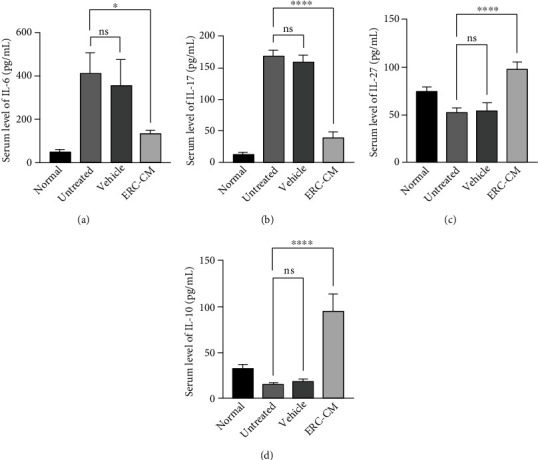
ERC-CM modulated the cytokine level in sera. Measurement of serum levels of IL-10, IL-27, IL-17, and IL-6 using the appropriate ELISA kits. IL-6 (a), IL-17 (b), IL-27 (c), and IL-10 (d) were shown, respectively. The *p* value was determined by one-way ANOVA. ^∗^*p* < 0.05, ^∗∗^*p* < 0.01, ^∗∗∗^*p* < 0.001, and ^∗∗∗∗^*p* < 0.0001.

To further assess the cytokine profile in mice, we performed separate assays from sera. Notably, the proinflammatory cytokines IL-6 and IL-17 were increased, and the anti-inflammatory cytokines IL-10 and IL-27 were decreased in the untreated group in the serum (Figures 7(a)–7(d)). After ERC-CM treatment, the above situation was reversed (IL-6: ERC-CM group *vs.* untreated group, *p* < 0.05; ERC-CM group *vs.* vehicle group, *p* < 0.05; IL-17: ERC-CM group *vs.* untreated group, *p* < 0.0001; ERC-CM group *vs.* vehicle group, *p* < 0.0001; IL-10: ERC-CM group *vs.* untreated group, *p* < 0.0001; ERC-CM group *vs.* vehicle group, *p* < 0.0001; and IL-27: ERC-CM group *vs.* untreated group, *p* < 0.0001; ERC-CM group *vs.* vehicle group, *p* < 0.0001). This suggests that therapeutic improvement of ERC-CM is associated with changes in cytokine levels in inflammatory mice.

### 3.8. ERC-CM Inhibited the Proliferation of Lymphocytes *In Vitro*

**Figure 8:**
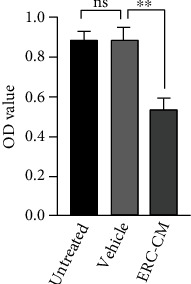
ERC-CM inhibits the proliferation of splenocytes *in vitro*. The proliferation of splenocytes in response to ConA was measured by using the CCK-8 kit. The *p* value was determined by one-way ANOVA. ^∗^*p* < 0.05, ^∗∗^*p* < 0.01, ^∗∗∗^*p* < 0.001, and ^∗∗∗∗^*p* < 0.0001.

To assess the immunosuppressive effects of ERC-CM *in vitro*, the splenocyte proliferation assay was performed. As shown in Figure 8, ERC-CM can inhibit the lymphocyte proliferation *in vitro* compared with the vehicle control (ERC-CM group *vs.* vehicle group, *p* < 0.01). ERC-CM significantly inhibits ConA-induced murine splenocyte proliferation.

## 4. Discussion

UC is a chronic inflammatory disease that plagues the world, and currently effective therapeutic strategies remain to be explored. In this study, acute colitis was successfully induced by drinking 3% DSS in BALB/c mice and identified with clinical symptoms. After receiving different treatments, some changes differentially exhibited in different groups; symptoms of colitis were significantly attenuated in the ERC-CM group. In addition, pathological changes, local inflammatory cell infiltration, and proinflammatory protein expression in the colon, as well as proinflammatory cytokine levels in sera, were significantly ameliorated in the ERC-CM-treated group when compared with those of vehicle and/or untreated groups, which indicated that ERC-CM can effectively exert therapeutic effects in the treatment of colitis. In addition, *in vitro* assays showed that ERC-CM effectively inhibited lymphocyte proliferation.

A growing number of studies suggest that MSCs exert their therapeutic effects depending not only on the way in which they come into contact with cells but also on their paracrine activity, mitochondrial transfer, etc. Paracrine function, on the other hand, is inevitably associated with the secretion of microvesicles and cytokines by MSCs [36, 37]. Various cytokines secreted by MSCs, such as insulin-like growth factor-1 (IGF-1), vascular endothelial growth factor (VEGF), IL-10, and TGF-*β*, are involved in tissue repair and the regulation of immune cells [38–40]. These extracellular vesicles and cytokines are contained in the CM *in vitro*. Compared with cell therapy, the use of CM can control the dose more accurately, and cell lines can be used for mass production without invasive extraction procedures for patients, which saves time and cost [41]. The CM acts in a more direct manner, unlike cells that may be captured by various host defense mechanisms. In addition, cytokines alone are expensive and may exaggerate the inflammatory response. Therefore, a combination of many different factors is the best way to treat [42]. Conditioned media can meet just such conditions. Innate and adaptive immunity is thought to play a major role in the pathogenesis of UC. Among them, T cells and macrophages are the main players [5], and it has been well documented that cells such as Th1 cells, Th17 cells, and M1 macrophages and the cytokines they secrete, such as IFN-*α*, IL-6, IL-17, and TNF-*α*, are involved in the pathogenesis of UC [43, 44]. It is well known that CD4^+^ T cells (Th1 cells, Th17 cells, and Tregs) are required for UC and their balance is essential for maintaining intestinal homeostasis. In the inflammatory environment, Th0 cells are converted to inflammatory Th1 and Th17 cells, contributing to driving the initial stage of colitis. The proinflammatory cytokines they release not only recruit neutrophils to the damaged areas but also promote more proinflammatory cytokine production through a negative feedback regulation mechanism. Tregs work as key effective suppressors in autoimmune diseases, not only suppressing the proliferation of Th0 cells *in vitro* and *in vivo* [45] but also exerting their function by producing the immunomodulatory cytokines, such as IL-10 and TGF-*β* [46]. In addition, previous evidence showed that purified Tregs prevented naive CD4^+^ T cell transfer-induced colitis in SCID mice [47]. As mentioned above, disruption of the dynamic balance between CD4^+^T cells is involved in the pathogenesis of colitis, and it is noteworthy that in patients with UC, Th17 cells are enriched and Tregs are scarce, and it has been well documented that the balance between Th17 cells and Tregs can be a potential regulatory target for improvement in colitis models [48, 49]. Macrophages make up a large proportion of intestinal immune cells, and they can initiate and coordinate the immune response against foreign bodies that have breached the epithelial barrier [50]. In inflammatory conditions, blood-derived monocytes/macrophages transform into a proinflammatory phenotype and produce large amounts of inflammatory cytokines such as IL-6 and IL-12, which are often described as classically activated macrophages (e.g., M1 macrophage) [51].

In the present study, percentages of Th1 cells, Th17 cells, and M1 macrophages are significantly increased in both the spleen and the colon in DSS-induced colitis when compared with normal mice, which are consistent with the results that Th1 cells, Th17 cells, and M1 macrophages may be involved in the pathogenesis of UC. In addition, percentages of Tregs in the spleen and the colon are increased in the ERC-CM-treated group when compared with untreated and/or vehicle groups, which implied the therapeutic effects of ERC-CM. *In vitro* experiments have also demonstrated that ERC-CM exerts a therapeutic effect by affecting lymphocyte populations. The mesenteric lymph nodes are an important tissue in response to local inflammation in the intestine and will be further analyzed in our future studies. The various pathogenic pathways of inflammatory bowel disease can interact with each other, and cytokines can be associated with any of them [52]. For example, IL-6 induces activation of the STAT3 signaling pathway, which regulates various genes involved in cell survival, cell migration, and apoptosis [53]. To determine whether ERC-CM ameliorates colitis by regulating cytokine levels, several cytokine levels were assayed. Our study showed that the proinflammatory cytokines IL-6, TNF-*α*, and IL-17 were decreased and the anti-inflammatory cytokines IL-27 and IL-10 were increased after ERC-CM treatment. It is worth noting that IL-27 was initially thought to be a proinflammatory cytokine, but the anti-inflammatory effects of IL-27 signaling have since been illustrated in many recent studies [54, 55]. It has been shown that IL-27 has a wide range of effects on Th1, Th2, and Th17 cells as well as Tregs [56]. This is also verified by the change in the ratio of various cells mentioned above. We therefore infer that the various changes occurring in colitis mice are associated with various cytokines.

We and others have previously demonstrated that ERCs play a therapeutic role in various diseases, including UC [18, 23]; however, whether ERC-CM as a cell-free therapy has a potent effect in attenuation of colitis remains unknown. Based on the previous work and to our knowledge, we are the first to explore the therapeutic effects of ERC-CM in colitis. The results showed very promising therapeutic prospects for ERC-CM. However, there are still some issues including the potential therapeutic mechanisms, and the strategy to improve the therapeutic effects of CM needs further investigation.

## 5. Conclusion

The results in this study have demonstrated that ERC-CM significantly attenuated colitis in mice, suggesting that ERC-CM could be used as a novel cell-free strategy for potential application in the treatment of inflammatory bowel diseases.

## Data Availability

All data included in this manuscript can be available.

## Abbreviations

ERCs: Endometrial regenerative cells
iNOS: Inducible nitric oxide synthase
TGF-*β*: Transforming growth factor-*β*
IGF-1: Insulin-like growth factor-1
DSS: Dextran sodium sulfate
DTT: Dithiothreitol
EDTA: Ethylene diamine tetraacetic acid
MSC: Mesenchymal stromal cells
IL-6: Interleukin-6
TNF-*α*: Tumor necrosis factor-*α*.
